# Nanorod Surface Plasmon Enhancement of Laser-Induced Ultrafast Demagnetization

**DOI:** 10.1038/srep15933

**Published:** 2015-10-30

**Authors:** Haitian Xu, Ghazal Hajisalem, Geoffrey M. Steeves, Reuven Gordon, Byoung C. Choi

**Affiliations:** 1 Department of Physics and Astronomy, University of Victoria, Victoria V8P 5C2, Canada; 2 Department of Electrical and Computer Engineering, University of Victoria, Victoria V8P 5C2, Canada

## Abstract

Ultrafast laser-induced magnetization dynamics in ferromagnetic thin films were measured using a femtosecond Ti:sapphire laser in a pump-probe magneto-optic Kerr effect setup. The effect of plasmon resonance on the transient magnetization was investigated by drop-coating the ferromagnetic films with dimensionally-tuned gold nanorods supporting longitudinal surface plasmon resonance near the central wavelength of the pump laser. With ~4% nanorod areal coverage, we observe a >50% increase in demagnetization signal in nanorod-coated samples at pump fluences on the order of 0.1 mJ/cm^2^ due to surface plasmon-mediated localized electric-field enhancement, an effect which becomes more significant at higher laser fluences. We were able to qualitatively reproduce the experimental observations using finite-difference time-domain simulations and mean-field theory. This dramatic enhancement of ultrafast laser-induced demagnetization points to possible applications of nanorod-coated thin films in heat-assisted magnetic recording.

A ferromagnetic (FM) thin film excited by femtosecond pulsed laser will undergo ultrafast loss of magnetic order (demagnetization) within several hundred femtoseconds, due to rapid energy transfer from thermalized electrons to the spin system. This is followed by a slower recovery (remagnetization) over the picosecond timescale as electrons equilibrate with the phonons, and eventually to complete cooling via nanosecond lattice diffusion^1^,^2^. The timescale of laser-induced ultrafast demagnetization is orders of magnitude below the limit imposed by conventional switching of magnetic order via magnetic field pulse (~100 ps^3^), and has been the focus of considerable research since its discovery in 1996^4^,^5^,^6^,^7^.

Orders-of-magnitude enhancement of electric fields, light absorption and scattering can occur as a result of the resonant excitation of conduction electrons – also known as localized surface plasmons (LSPs), in a metal nanoparticle (NP) under illumination^8^. The resonant frequency is highly sensitive to NP geometry and environment^9^, and is tunable from visible to near infrared. With recent advances in the synthesis of highly monodisperse colloidal noble metal NPs^10^, plasmon NPs have been the subject of investigation in a wide range of plasmonic applications, including cancer diagnosis and therapy, remote delivery and photothermal imaging^11^,^12^. Furthermore, an increasing number of ‘magnetoplasmonic’ studies have emerged, focusing on combining magnetic and plasmonic functionalities^13^,^14^. Of particular relevance to information storage is the on-going development of heat-assisted magnetic recording (HAMR) technology, which uses a near-field plasmon NP transducer to resonantly focus laser light onto a magnetic recording medium, temporarily reducing its coercivity through heat generation, thus lowering the write-field requirement^15^,^16^,^17^,^18^. A primary issue associated with current HAMR prototypes is the accelerated thermal degradation of the transducer due to thermal cycling, which limits their long-term reliability^19^,^20^. Several solutions have been proposed, including two-stage heating of the recording medium^21^ as well as the ongoing search for alternative, low-loss plasmon materials^22^. Nevertheless, thermal degradation of the near-field transducer remains a major concern.

In this work, we investigate the improvement in laser-induced demagnetization signal through LSP-enhanced localized heating. We show that, by coating FM thin films with gold nanorods (AuNRs) dimensionally-tuned to support LSPs near the peak laser frequency, the demagnetization can be increased by over 50% for ~4% AuNR coverage, without increasing the laser fluence. The AuNR coating functions in a similar capacity to NP transducers in HAMR recording heads, and the arrangement offers a possible solution to the thermal degradation problem in current HAMR prototypes by uniformly distributing or patterning the plasmon NPs across the surface of the recording medium, thus subjecting individual AuNRs to a small fraction of the overall thermal cycling.

## Results

**Figure 1:**
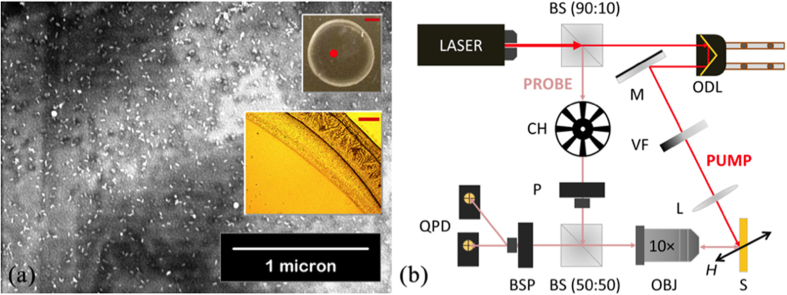
(**a**) SEM image showing drop-evaporated AuNR distribution on film surface. Top inset: drop-evaporated spot. Red dot indicates the approximate position where the SEM image was recorded. Scale bar = 1 mm. Bottom inset: bright-field image taken at the edge of the drop-evaporated spot under 20× objective, showing details of the highly non-uniform AuNR aggregation. Scale bar = 50 *μ*m. (**b**) Pump-probe setup for measuring transient magnetization. Abbreviations: BS: beam splitter, BSP: polarizing beam-splitter, CH: chopper, *H*: external field, L: lens, M: mirror, ODL: optical delay line, OBJ: objective, P: polarizer, QPD: quadrant photodiode, S: sample, VF: variable filter.

Colloidal 10 nm × 41 nm AuNR was drop-evaporated onto 20 nm ferromagnetic Ni_80_Fe_20_ (permalloy) thin films to achieve an average density of 96 AuNR/*μ*m^2^ (a total AuNR cross-sectional area of 39360 nm^2^ per *μ*m^2^, or 3.94% areal coverage), with a standard deviation of 41 AuNR/*μ*m^2^, as shown in the SEM image in Fig. 1(a). Due to capillary pressure, there is a highly non-uniform aggregation of AuNR at the edge of the evaporated spot (Fig. 1(a), lower inset), and care was taken during subsequent experiments to avoid this edge formation. To independently verify the plasmon resonance effect of the AuNR coating, dark-field scattering spectra of the thin film samples were obtained with a white light source at 70^o^ incidence using a setup described elsewhere^23^,^24^. Molar extinction coefficient of the colloidal solution was measured using the Beer-Lambert law^25^. A stroboscopic pump-probe magneto-optic Kerr effect setup in the polar configuration, schematically shown in Fig. 1(b), was used to measure time-resolved out-of-plane magnetization, *M*_*z*_ under the illumination of a femtosecond Ti:sapphire pulsed laser, tuned to the plasmon frequency of the AuNRs^26^.

**Figure 2:**
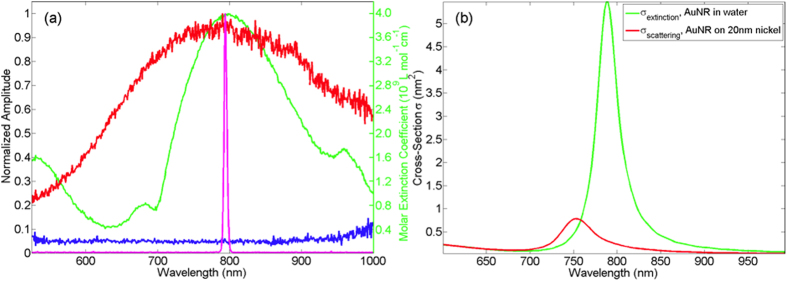
(**a**) Dark-field scattering spectra of AuNR-coated (red) and uncoated (blue) permalloy films (arbitrary units). Magenta: Ti:sapphire laser spectrum (arbitrary units). Green: molar extinction coefficient spectrum of the colloidal AuNR solution (unit: 10^9^ L mol^−1^ cm^−1^, right y-axis). (**b**) Simulated scattering spectra of 10 × 40 nm AuNR in water (red) and on 20 nm nickel (green). Material data taken from ref. 30,31.

Figure 2(a) shows the dark-field scattering spectra of the AuNR-coated sample (red) normalized against the regular permalloy film (blue), as well as the molar extinction coefficient spectra of the colloidal AuNR solution (green) and the Ti:sapphire laser (magenta). The AuNR-coated thin film shows a distinct spectral peak slightly below 800 nm, close to the central wavelength of the Ti:sapphire laser. The uncoated sample by comparison does not exhibit any discernable peaks over the entire (500–1000 nm) frequency range. The spectral peak of the AuNR-coated sample exhibits a slight blue-shift and significant broadening compared to the colloidal solution, which may be attributed to the modification of LSP due to interaction between nanorods and the close proximity of the permalloy substrate^27^,^28^. Simulations using a commercial finite-difference time-domain (FDTD) package (Lumerical Solutions, www.lumerical.com) reveals a qualitative agreement with experimental observations (Fig. 2(b)).

**Figure 3:**
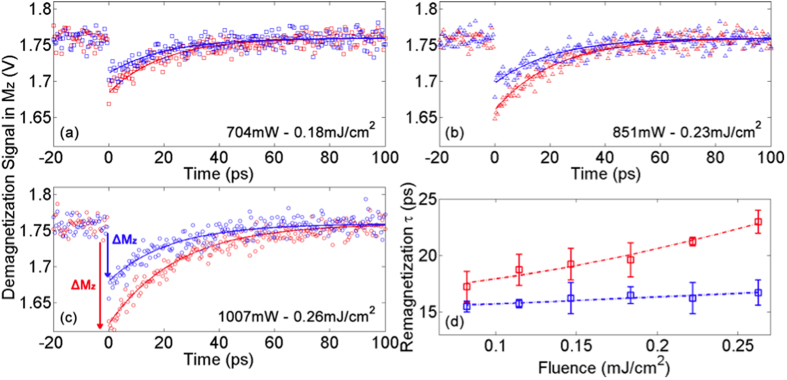
Laser pulse-induced ultrafast demagnetization measured with (red) and without (blue) AuNR coating at pump fluences of (**a**) 0.18 mJ, (**b**) 0.23 mJ and (**c**) 0.26 mJ. Time-step used = 500 fs. Solid lines are single exponential fits. (**d**) Characteristic remagnetization time τ versus laser fluence for samples with (red) and without (blue) AuNR. Lines serve as visual guides.

Laser-induced ultrafast demagnetization signals in *M*_z_ were recorded for a range of pump fluences, *F* ≈ 0.08 – 0.26 mJ/cm^2^ (315–1007 mW). Selected results are shown in Fig. 3 for uncoated (blue) and AuNR-coated (red) permalloy samples for different pump fluences. The characteristic sub-picosecond demagnetization, the subsequent picosecond remagnetization as well as the increase in demagnetization signal with fluence are in general agreement with existing literature^29^,^30^,^31^,^32^,^33^. A long scan range (~100 ps with 500 fs time-steps) was used in order to capture the slow remagnetization process. This temporal regime is orders of magnitude longer than the subpicosecond pulse duration and thermalization time^34^, therefore the remagnetization process can be adequately described by a single exponential expression^35^, *M*(*t*) = *C*_1_exp(–*t*/*τ*) + *C*_2_, where the characteristic remagnetization time *τ* is quantitatively defined as the time at which magnetization recovers by 63%.

Most interestingly, the results in Fig. 3 reveal an increase in ∆*M*_*z*_ in the AuNR-coated sample compared to the uncoated sample at the same pump fluence. Plots of characteristic remagnetization times, *τ* against pump fluence are shown in Fig. 3(d). τ increases with increasing pump fluence, due to the increasing loss of magnetic correlation with increasing temperature^36^. For the AuNR-coated sample, *τ* is longer at all pump fluences, which complements the demagnetization data and indicates a higher ‘effective temperature’ in the LSP-enhanced thin films.

**Figure 4:**
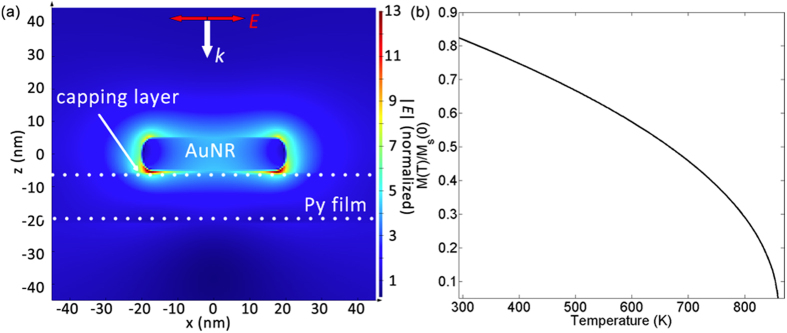
(**a**) Cross-sectional electric field |***E***| profile for 10 nm × 40 nm AuNR on 15 nm permalloy with 1 nm spacer layer at plasmon resonance, calculated using Lumerical Solutions. Simulation mesh size = 0.5 nm in the vicinity of the AuNR. Dotted lines represent the boundaries of the permalloy (Py) film. (**b**) Temperature-dependence of magnetization for permalloy, calculated using *M*(*T*) ~ (1 − *T*/*T*_*C*_)^*β*^.

In order to better interpret these results, we performed first-order calculations of the localized AuNR field enhancement, and the subsequent heat generation and demagnetization in a FM thin film with 4% AuNR coverage. Using experimentally measured laser fluences *F*, the averaged temperature increase in the regular FM substrate after pulse absorption ∆*T*_FM_ was estimated using


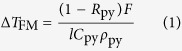


where *R*_py_ is the experimentally measured reflectance (scattering is ignored for the film sample), laser penetration depth *l* = 15 nm and *C*_py_ and *ρ*_py_ are the heat capacity and density of permalloy-80, taken from ref. 37. Given the scan range and temporal resolution (100 ps/500 fs) of the current investigation, thermalization (<1 ps^34^,^35^) was considered instantaneous. Intricacies resulting from the interplay between the various demagnetization and thermalization timescales are left for a future study. For FM film coated with AuNR, enhancement in heating originates from the enhanced electric field |***E***| inside the FM substrate at plasmon resonance and can be calculated as follows^38^:


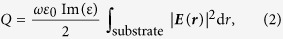


where *Q* is the heat generation, *ω* is the angular frequency of the laser, and *ε*_*0*_ and *ε* are the vacuum and relative permittivity of the FM film, respectively. For a given incident electric field amplitude and polarization, *E*(r), and hence *Q* and ∆*T*_FM_, can be computed using Lumerical Solutions. To take into account random orientations of the AuNRs with respect to the linearly polarized pump pulse in our experiment, an extra factor of 0.5 was applied to the *Q* calculated for the case where AuNR is aligned in parallel to electric field polarization (Fig. 4(a)), in order to better estimate an ‘average’ temperature increase. The capping layer around the colloidal AuNR (cetyltrimethylammonium bromide (CTAB)) was modeled as a 1 nm spacer layer with refractive index equal to that of air, which has been previously shown to be a reasonable assumption for thin CTAB layers in the near-field^39^. As a result of the field enhancement *inside* the AuNR, they act as sources of heat^40^ and must be taken into account. COMSOL Multiphysics (version 4.3.0.233) was used to investigate conductive heat transfer from AuNRs to the FM layer using initial AuNR temperatures calculated from equation (2). The *additional* temperature increase in the FM layer in this case was found to be negligible over the demagnetization timescale (~ps), due to the presence of the CTAB ligand and the small area of contact between the AuNR and the FM film. Using calculated values of ∆*T*_FM_, ∆*M*_*z*_ at different fluences were found for both regular and AuNR-coated permalloy substrates using the temperature-dependence of magnetization, *M*(*T*) ~ (1 – *T*/*T*_*C*_)^*β*^ (Fig. 4(b)), where Curie temperature *T*_*C*_ = 860 K for permalloy^41^ and critical exponent *β* = 0.5 from mean-field theory^42^.

**Figure 5:**
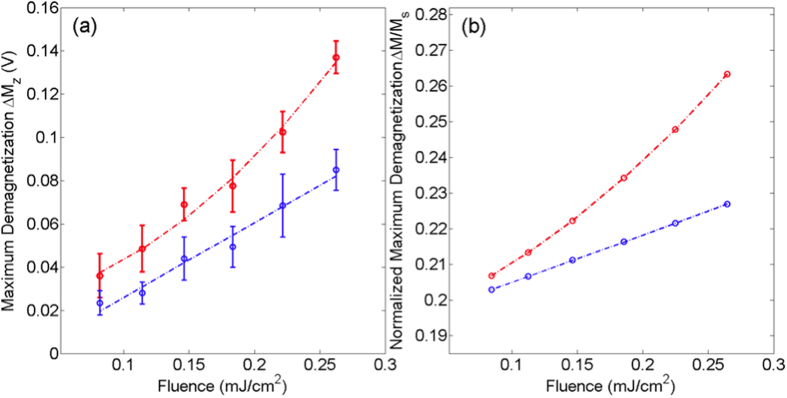
Fluence dependence of demagnetization for AuNR-coated (red) and uncoated (blue) samples. (**a**) experimental results; (**b**) results from model calculations. Lines serve as visual guides.

The fluence dependence of demagnetization from both experiment and modeling are presented in Fig. 5. There is a consistent enhancement in ∆*M*_*z*_ for AuNR-coated samples over the entire experimental fluence range. At higher pump fluences, this enhancement becomes more significant, and ∆*M*_*z*_ for the AuNR-coated sample exhibits divergent behavior due to the increased curvature *M*(*T*) at temperatures approach *T*_*C*_ (Fig. 4(b)). At the highest experimental pump fluence (*F* ~ 0.26 mJ/cm^2^), ∆*M*_*z*_ in the AuNR-coated sample is increased by ~60% compared to the uncoated sample. By extrapolating the linear fit in Fig. 5(a), it was found that the uncoated sample would require a pump fluence *F* ~ 0.42 mJ/cm^2^ in order to achieve the same ∆*M*_*z*_ as the AuNR-coated sample at *F* ~ 0.26 mJ/cm^2^. Despite its simplicity, our model calculations were able to qualitatively reproduce the experimental results (Fig. 5(b)). We note that the simulation does not take into account the variation in density and orientation of the drop-evaporated nanorods, or thermal diffusion to the surroundings, leading to an over-estimation in demagnetization.

In conclusion, we demonstrated the LSP-enhancement of laser-induced ultrafast demagnetization on permalloy thin films coated with drop-evaporated colloidal AuNRs supporting LSP near the peak laser frequency. Compared to the uncoated sample, an increase in demagnetization signal of >50% was observed in the AuNR-coated samples with 4% AuNR coverage, which increases with increasing laser fluence. Despite the low areal coverage of AuNR, LSP-enhanced electric-field intensity in the permalloy substrate in the vicinity of AuNR contributes disproportionately to the overall demagnetization signal. We were able to qualitatively reproduce the experimental results with FDTD modeling and mean-field theory.

## Methods

### Sample preparation

20 nm films of ferromagnetic Ni_80_Fe_20_ (permalloy) with 5 nm titanium adhesion were deposited using electron-beam evaporation in 1.0 × 10^−6^ Torr vacuum at 1 Å/s on native-oxide coated (110) Si wafers. The film thickness was verified post-deposition with a spectroscopic ellipsometer (J.A. Woollam Alpha-SE), and found to be 20.348 nm, with an average roughness of 0.327 nm. 10 *μ*L of commercial monodisperse 10 nm × 41 nm colloidal AuNR solution (Nanopartz, 7.24 × 10^11^/mL, CTAB-capped) with longitudinal plasmon resonance wavelength *λ* = 808 nm was drop-cast onto the film surface and allowed to evaporate under closed ambient conditions to minimize density variation and band formation^43^. AuNR density and variability were measured by visually inspecting 3 × 3 *μ*m^2^ areas of the SEM images of the sample.

### Experimental methods

In order to trigger ultrafast demagnetization, a 76 MHz Ti:sapphire laser (Coherent Mira 900) pump beam with 795 nm central wavelength and 135 fs pulse duration was focused on a *r* ≈ 40 *μ*m spot, for a maximum pulse fluence *F* ≈ 0.33 mJ/cm^2^ (~16 nJ/pulse). This fluence is sufficiently large to induce measurable demagnetization, but does not melt the AuNRs or cause a significant, permanent temperature increase during measurement^44^. The ratio of pump to probe intensity at sample surface was >20:1. The probe beam was modulated at 300 Hz using a chopper and was focused on an overlapping area with *r* ≈ 10 *μ*m with the help of a CCD camera. When measuring AuNR-coated samples, care was taken to position the probe beam over areas on the sample with close to average AuNR density. The delay between pump and probe was mechanically controlled via a 220 mm (1466 ps) optical delay line (Thorlabs ODL220-FS), with a delay precision of ±0.67 fs. An external field *H* = 140 kA/m inclined at 30^o^ to the normal was applied to create a canted, out-of-plane saturation magnetization. Transient changes in *M*_*z*_ in terms of Kerr rotation *θ*_*K*_, averaged over the 10 *μ*m probe spot in the Py film, were measured using a balanced photodiode optical bridge detector, which isolates the Kerr and the reflectivity signals^45^. The detector system consists of a polarizing beam splitter (Wollaston prism) and a pair of silicon quadrant photodiodes (Thorlabs PDQ80A) with lock-in amplification referenced at the modulation frequency of the probe beam. The difference between two photodiode signals is proportional to the Kerr rotation. The experiment was carried out at room temperature.

## Additional Information

**How to cite this article**: Xu, H. *et al.* Nanorod Surface Plasmon Enhancement of Laser-Induced Ultrafast Demagnetization. *Sci. Rep.* **5**, 15933; doi: 10.1038/srep15933 (2015).
